# Benefits and Harms of Extending the Duration of Dual Antiplatelet Therapy after Percutaneous Coronary Intervention with Drug-Eluting Stents: A Meta-Analysis

**DOI:** 10.1155/2014/794078

**Published:** 2014-03-02

**Authors:** Chun Shing Kwok, Heerajnarain Bulluck, Alisdair D. Ryding, Yoon K. Loke

**Affiliations:** ^1^Institute of Cardiovascular Sciences, University of Manchester, Manchester Royal Infirmary, Manchester M13 9WL, UK; ^2^Norfolk and Norwich University Hospital, Colney Lane, Norwich NR4 7UY, UK; ^3^Norwich Medical School, University of East Anglia, Norwich Research Park, Norwich NR4 7TJ, UK

## Abstract

*Background.* The optimal duration of dual antiplatelet therapy (DAPT) after percutaneous coronary intervention (PCI) is unclear. *Methods.* We conducted a systematic review and meta-analysis of randomized controlled trials evaluating risk of adverse events in participants receiving different durations of DAPT following insertion of drug-eluting stents. *Results.* Five trials were included, but only four had data suitable for meta-analysis (*n* = 8,231 participants). No significant increase in the composite endpoint of death and nonfatal myocardial infarction was observed with earlier cessation of DAPT in any instance when compared to longer durations of DAPT (RR 0.64 95% CI 0.25–1.63 for 3 versus 12 months, RR 1.09 95% CI 0.84–1.41 for 6 versus 12 months and, RR 0.64 95% CI 0.35–1.16 for 12 versus 24 months). Pooled results showed a significantly lower risk of major bleeding (RR 0.48 95% CI 0.25–0.93) and total bleeding (RR 0.30 95% CI 0.16–0.54) for shorter compared to longer duration of DAPT. Subgroup analysis based on age, prior diabetes, and prior ACS failed to show any group where longer durations were consistently better than shorter ones. *Conclusions.* There are no cardiovascular or mortality benefits associated with extended duration of DAPT, but the risk of major bleeding was significantly lower with shorter lengths of therapy.

## 1. Introduction 

Concerns over the possibility of early coronary stent thrombosis have led to calls for longer durations of dual antiplatelet therapy (DAPT) following drug-eluting stent implantation. Whilst prolonged courses of therapy may possibly reduce the risk of thrombotic complications, this strategy is offset by the risk of bleeding complications. Clinical guidelines vary between North America and Europe. The American Heart Association/American College of Cardiology recommend that clopidogrel and aspirin therapy should be extended to at least 12 months after DES implantation if patients are not at high risk of bleeding [1]. Conversely the European Society of Cardiology recommends 6 to 12 months of DAPT for patients following elective PCI and DES implantation or 12 months in acute coronary syndromes [2]. These guidelines are largely based on observational studies which show that early discontinuation of clopidogrel therapy is predictive of stent thrombosis [3–5]. One prospective cohort study of 2,229 patients who had received DES found that 29% of participants who discontinued antiplatelet therapy prematurely had a thrombotic event [5]. Another observational study compared patients who had discontinued clopidogrel therapy at 6 months to those who continued therapy and a 3% of increase in death and 4% of increase in MI or death were associated with discontinuation of therapy [6].

Since these observational studies were performed, several randomized controlled trials have been conducted to address this question [6–10]. A recent meta-analysis was performed by Cassese et al. which summarized the findings of these trials [11]. However, this meta-analysis did not explicitly present results according to differences in cutoff time for duration of DAPT. The reviewers did not look at consistency of findings across different patient groups (such as the elderly or those with diabetes mellitus), and the meta-analysis may have lacked power overall because it considered only specific individual endpoints rather than a composite of clinically important adverse events such as death or myocardial infarction.

The aim of our study is to perform a meta-analysis of high quality randomized controlled trials that evaluated risk of mortality and thrombotic events associated with different durations of DAPT after PCI with drug-eluting stents.

## 2. Methods

### 2.1. Eligibility Criteria

We selected parallel group randomized controlled trials involving dual antiplatelet therapy (aspirin plus any of the following agents: clopidogrel or ticlopidine or prasugrel or ticagrelor) for a specified duration following coronary stenting with the comparator arm being dual antiplatelet therapy for a different duration. While the main focus was on patients treated with drug-eluting stents, we also accepted trials that had a mixture of patients with bare metal and drug-eluting stents. We excluded trials that compared different stents, or different antiplatelet agents rather than different durations of antiplatelet therapy after coronary stenting.

### 2.2. Search Strategy

We searched MEDLINE and EMBASE through OvidSP using the Haynes optimized search strategy (Health Information Research Unit, McMaster University) [12]. The exact search strategy is shown in Appendix  1 (see Appendix  1 in Supplementary Material available online at http://dx.doi.org/10.1155/2014/794078). We also checked the bibliographies of included trials for any relevant studies. In addition, we used the PubMed automated updates for new articles up to August 2013.

### 2.3. Study Selection and Data Abstraction

Two reviewers (CSK and HB) independently and in duplicate assessed titles and abstracts and excluded those that were clearly not relevant. The reviewers then went on to obtain full-text versions of potentially suitable articles for detailed evaluation against the eligibility criteria.

Following discussion and full agreement on the included and excluded studies, the two reviewers independently extracted data from relevant studies. The data extraction was then checked by the other authors (YKL and ADR) and full consensus was reached after resolving any discrepancies through discussion and further review of the manuscripts.

Our primary outcome of interest was a composite of all-cause death and nonfatal myocardial infarction. We also considered individual endpoints of myocardial infarction, stroke, cardiovascular death, all-cause mortality, stent thrombosis, and need for revascularization.

We aimed to evaluate adverse events including thrombolysis in myocardial infarction major bleeding, as well as specific subcategories of gastrointestinal bleeding and intracranial hemorrhage.

### 2.4. Study Characteristics and Quality Assessment

Two reviewers (CSK and HB) extracted data on study characteristics, which was then checked by the other reviewers (AR and YKL). We recorded the study design, duration of DAPT exposure, number of participants, duration of followup, outcomes evaluated, outcome events, PCI procedural data, angiogram results, patient selection criteria, compliance with medication, and doses of antiplatelet used in the RCTs.

Quality assessment was conducted based on the recommendations of the Cochrane handbook of systematic reviews [13] which included consideration of randomization sequence generation, allocation concealment, blinding of participants, personnel and outcome, incomplete or selective outcome reporting, and publication bias. We aimed to produce a funnel plot if there were >10 included studies with no evidence of statistical heterogeneity.

### 2.5. Quantitative Data Synthesis

RevMan 5.021 (Nordic Cochrane Center) was used to conduct fixed-effect meta-analysis for the pooled relative risks (RR), with 95% confidence intervals for dichotomous outcomes. The main analysis was on an intention to treat basis, and all reported *P* values are two-sided, with significance set at *P* less than 0.05. Statistical heterogeneity was assessed using *I* ^2^ statistic, with *I* ^2^ values of 30–60% representing a moderate level of heterogeneity [14].

We aimed to perform prespecified subgroup analysis based on nature and duration of antiplatelet therapy, type of stent, and specific patient populations such as the elderly or those with diabetes mellitus. Risk ratios were pooled using the inverse variance method for specific patient subgroups in the trials.

## 3. Results

### 3.1. Study Selection, Design, and Methodology

**Figure 1:**
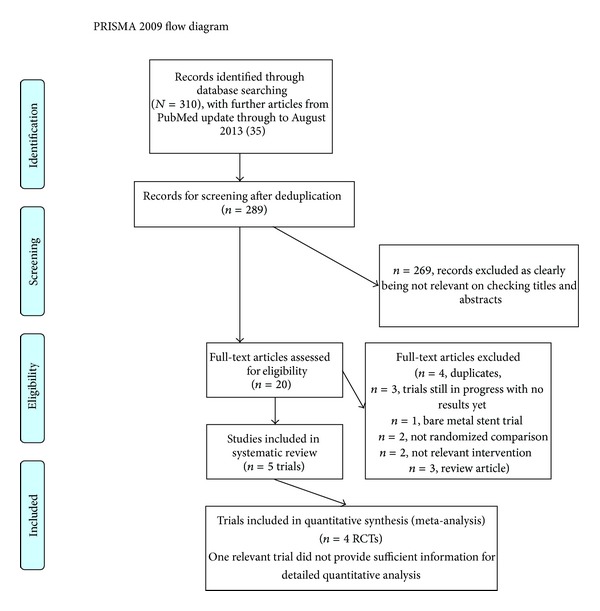
Flow diagram of study selection (from [15, 16]).

Five studies met the inclusion criteria (4 open label RCTs [6–9] and 1 double-blind RCT [10]) and the process of study selection is shown in Figure 1. Four studies were included in the meta-analysis as one of the studies (presented as a conference abstract) provided insufficient information to allow detailed quantitative evaluation.

The four trials included a total of 8,231 participants and these trials were based in South Korea and Italy between 2006 and 2010. The definition of short and long duration of studies varied from 3 to 12 months for the shorter duration and from 12 to 36 months for the longer duration and participants in the trials were followed up for up to 2 years. One trial compared 3 months versus 12 months, two compared 6 months versus 12 months, and one trial evaluated 12 months versus 24 months. While all the trials compared aspirin and clopidogrel, the dose was consistent at 75 mg for clopidogrel across all trials but the dose of aspirin varied from 100 to 200 mg. The types of stents used varied among the trials, but they all used one or more of the following stents: everolimus-eluting stent, sirolimus-eluting stent, Endeavor zotarolimus-eluting stent, Resolute zotarolimus-eluting stent, paclitaxel-eluting stents, and bare metal stent. All four studies reported stent thrombosis, myocardial infarction/acute coronary syndrome, all-cause mortality, and major bleeding event.

These studies along with the design, year of study, definition of short and long duration, total number of participants in each group, duration of follow-up, and outcomes evaluated are shown in Table 1. The patient's selection criteria and demographics, comorbidities, angiography results, PCI procedural information, study outcomes, and compliance with medications are shown in Appendices  3–7. The rates of thrombotic events and bleeding events are shown in Tables 2 and 3, respectively.

### 3.2. Quality Assessment

The quality of studies is shown in Appendix  2. Randomization was considered adequate for most trials, but some degree of loss to follow-up was noted. Although the four studies included in the meta-analysis were open-label, the adverse events were independently adjudicated by blinded observers.

We did not test for publication bias as there were too few trials, but our search does include conference abstracts which reduces the risk of publication bias.

### 3.3. Pooled Analysis of Death and Nonfatal Myocardial Infarction for Shorter as Compared to Longer Duration of DAPT

**Figure 2:**
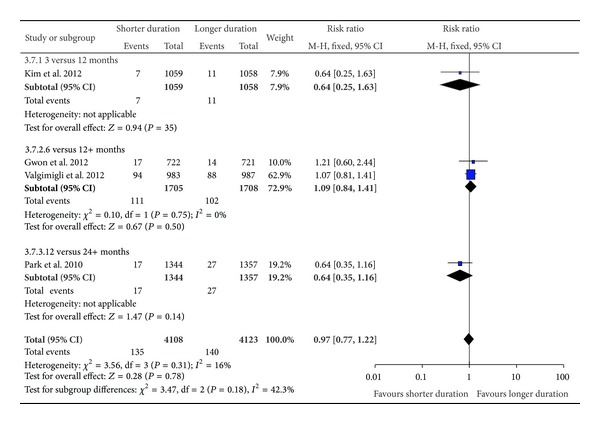
Risk of composite endpoint of death and myocardial infarction.

For the composite measure of all-cause mortality or nonfatal MI (Figure 2), no significant increase in risk with shorter DAPT duration was observed for the study comparing 3 versus 12 months (RR 0.64 95% CI 0.25–1.63), two studies of 6 versus 12 months (pooled RR 1.09 95% CI 0.84–1.41), and one study of 12 versus 24 months (RR 0.64 95% CI 0.35–1.16). Overall, the pooled risk ratio for mortality or nonfatal myocardial infarction with shorter DAPT across the four trials was 0.97 (95% CI 0.77–1.22). We found little statistical heterogeneity between trials (*I* ^2^ = 18%). Although the trials varied in their cut-off time for DAPT discontinuation, subgroup testing did not demonstrate any significant differences (*P* = 0.18) in the results of the three datasets when stratified according to duration of DAPT.

### 3.4. Pooled Analysis of Risk of Thrombotic Events or Mortality for Shorter as Compared to Longer Duration of DAPT

**Figure 3:**
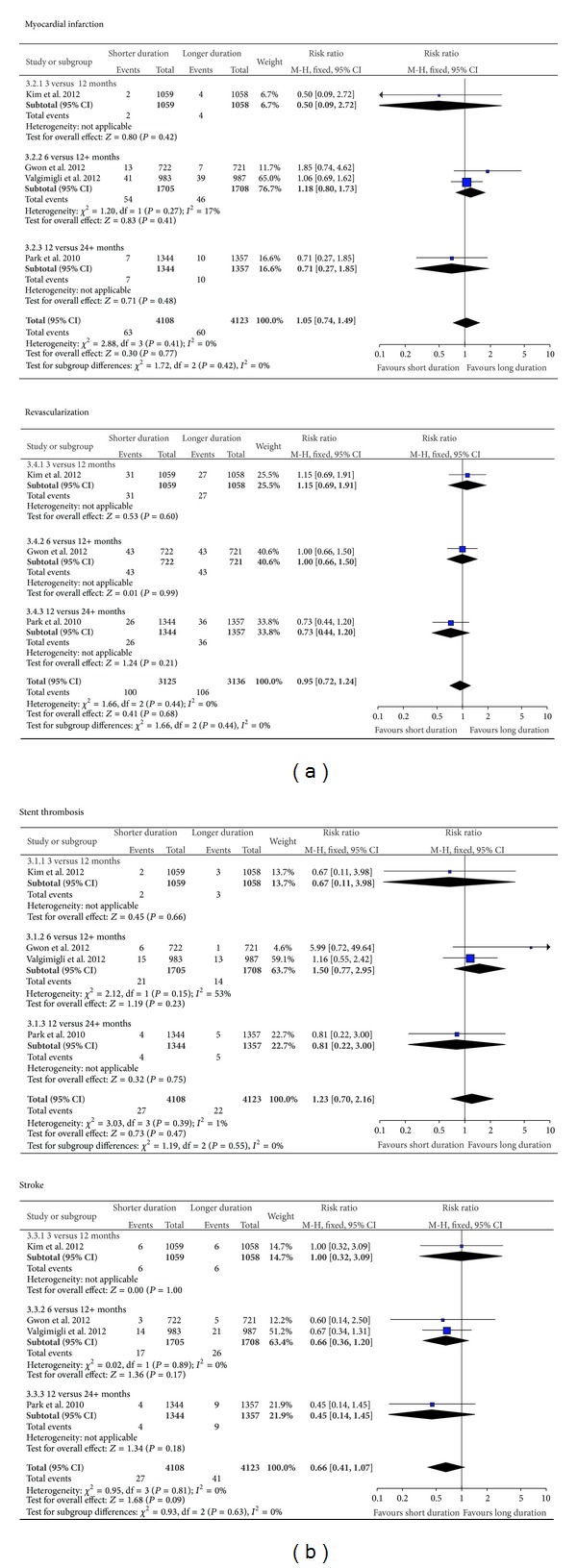
Risk of stent thrombosis, myocardial infarction, stroke, and revascularization.

The results for the pooled risks of stent thrombosis, myocardial infarction, stroke revascularization, cardiovascular death, and overall mortality are shown in Figure 3. Again, subgroup testing did not demonstrate any significant differences in the results of individual endpoints from the three datasets when stratified according to duration of DAPT.

### 3.5. Pooled Analysis of Risk of Bleeding for Shorter as Compared to Longer Duration of DAPT

**Figure 4:**
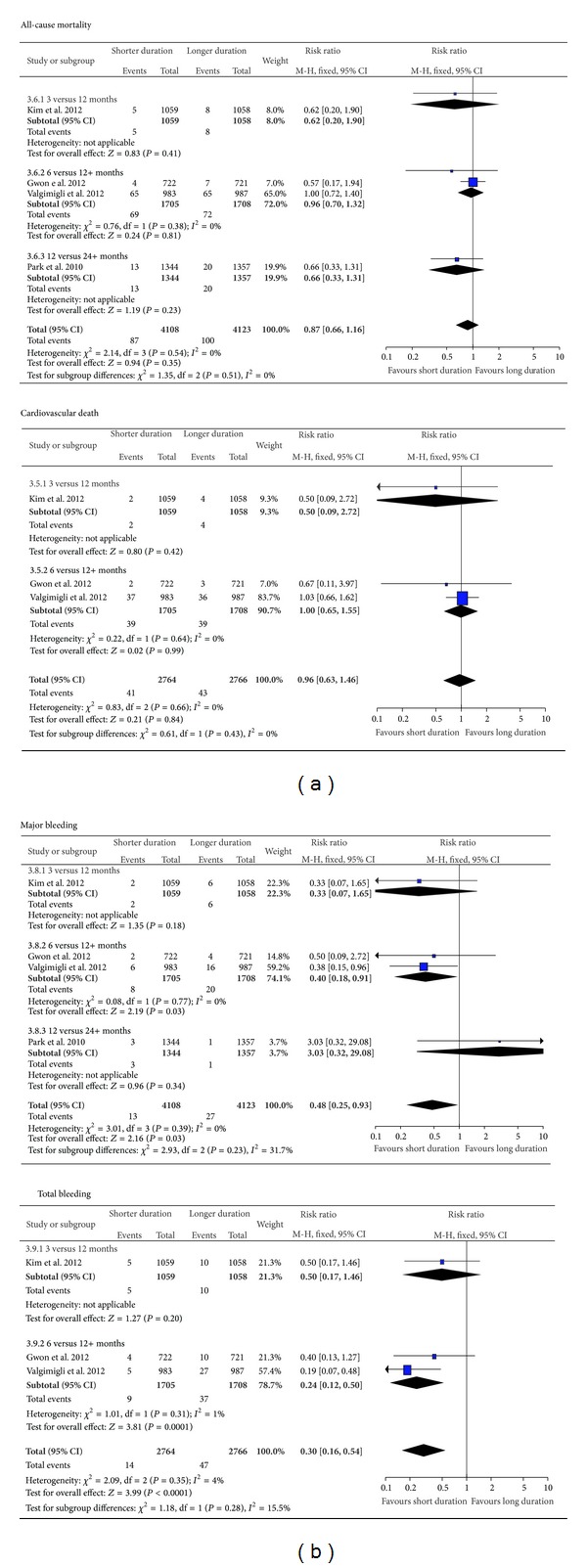
Risk of mortality and bleeding.

There was a significantly reduced risk of major bleeding with early discontinuation of antiplatelet therapy comparing 6 and >12 months (RR 0.40 95% CI 0.18–0.91) (Figure 4). The pooled result showed significantly lower risk of major bleeding (RR 0.48 95% CI 0.25–0.93) for shorter versus longer duration, with no significant differences found amongst the subgroups stratified according to duration of DAPT. A significantly lower risk of total bleeding was observed in the comparison of 6 versus 12+ months (RR 0.24 95% CI 0.12–0.50) and in the pooled results across all four trials (RR 0.30 95% CI 0.16–0.54).

### 3.6. Risk of Adverse Outcomes with Specified Subgroup with Continued and Discontinued Antiplatelet Therapy

**Figure 5:**
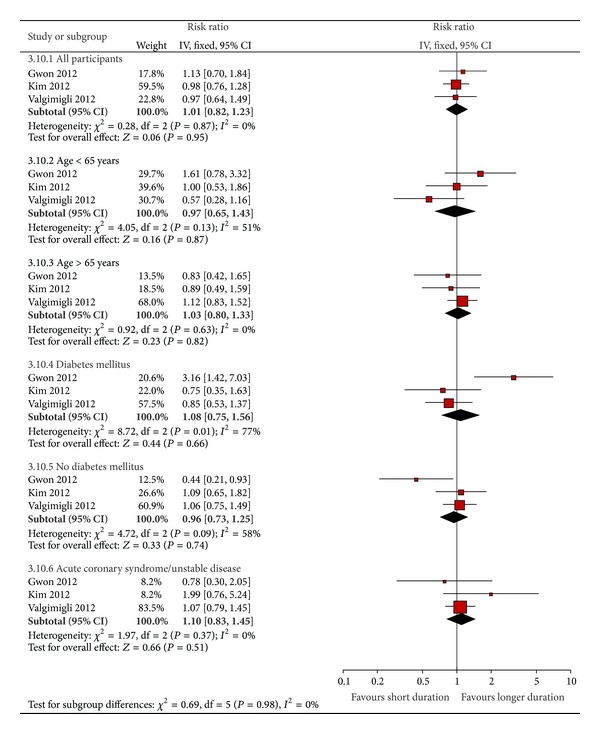
Risk of adverse primary outcomes in specific subgroups.

The results of additional subgroup analyses for three trials are shown in Figure 5. Here, the adverse primary outcome (as specified by trial investigators) for all participants as well as subgroups of participants was analyzed from three of the trials that provided relevant data. The risk of adverse outcomes with shorter duration of DAPT did not differ significantly in subgroups of patients with age >65 years (RR 1.03 95% CI 0.80–1.33) or those with age <65 years (RR 0.97 95% CI 0.65–1.43). Although one trial found that the subgroup of patients with diabetes mellitus appeared to have a significantly greater risk of adverse outcomes with shorter DAPT [6], this finding was not replicated in the other two trials [7, 9]. Overall, there was no robust evidence of significantly increased or decreased risk of adverse outcomes with DAPT in those with or without diabetes mellitus, with moderate to substantial heterogeneity detected (*I* ^2^ > 50% in the analyses). For the subgroup of patients with prior acute coronary syndrome or unstable disease, no significant difference in rates of adverse primary outcomes was observed between shorter and longer durations of DAPT (RR 1.10 95% CI 0.83–1.45). Test for subgroup difference (*P* = 0.98) amongst all the above-mentioned patient categories did not identify any subset where shorter duration of DAPT was associated with significantly more risk of adverse outcomes.

### 3.7. Additional Consideration of Study by Hu and Wang

One study by Hu et al. was not included in the pooled analysis, but its findings are consistent with those of our meta-analysis [10]. This trial of 216 participants in China randomized patients to 12 months or ≥36 months of dual antiplatelet therapy and found that there was no difference between the groups for mortality, myocardial infarction, stent thrombosis, or composite outcomes.

## 4. Discussion

The optimal duration of DAPT after DES implantation is a balance between the thrombotic and hemorrhagic risks. Our analysis of randomized data demonstrates that extending the period of DAPT does not significantly reduce the risk of thrombotic harm and mortality when compared to shorter durations following PCI with drug-eluting stents. Overall, the upper bounds of the 95% confidence interval for relative risk of death or nonfatal MI with shorter durations were 1.22, indicating that patients are unlikely to face an increased risk beyond 22% as compared to longer durations of DAPT. The risk of hemorrhagic complications clearly increased with prolonged durations of DAPT, and overall 12 months or less of DAPT was associated with least harm. However, it is not possible to recommend a more precise duration of therapy based on current data.

The apparent absence of cardiovascular harm with shorter durations was consistently noted in different subgroups stratified by duration of DAPT, individual endpoints, or patient characteristics. Although Gwon et al. [6] suggest that patients with diabetes mellitus were at significantly higher risk of myocardial infarction and target vessel revascularization in the short DAPT group, our subgroup analysis did not consistently demonstrate any impact of age, presence of diabetes mellitus, and ACS on the adverse primary outcomes in both short and long DAPT groups.

Stent thrombosis may have catastrophic consequences and suboptimal DAPT duration plays an important part [3, 17, 18]. Earlier RCTs during the bare metal stent (BMS) era [19–21] demonstrated that longer durations of DAPT after PCI reduced ischemic events. With the increasing use of DES, several observational studies have reported that early discontinuation of clopidogrel therapy is predictive of stent thrombosis [4, 5, 17, 18]. However, none of the studies reported bleeding complications, so they may provide an unbalanced view of the risk/benefit of DAPT. The current guidelines on DAPT duration [1, 2, 22] are based on these observational studies, which means they have inherent limitations.

A meta-analysis of 22 RCTs [23] comparing BMS and DES showed no difference in risk of acute ischemic events with DES, and the newer generations of DES have consistently shown lower rates of late and very late ST [24, 25]. A recent network meta-analysis has in fact shown that everolimus-eluting stents (EES) might have lower risk of ST than BMS within 2 years [26]. These findings have supported the notion that shorter duration of DAPT might be safe and adequate and this was demonstrated in 2 recent meta-analyses by Zhang et al. [27] and Ba et al. [28] derived predominantly from observational studies. The 4 RCTs [6–9] we included in our study were specifically designed to test this hypothesis and this makes our meta-analysis more robust.

Our results build upon the findings of the recent meta-analysis conducted by Cassese et al. [11]. We were able to include the composite outcome of mortality and myocardial infarction and this gives our study greater power and subsequently narrower confidence intervals for the pooled estimate. For instance, Cassese's meta-analysis focused on overall mortality as their main endpoint, and the 95% confidence intervals indicate that they were unable to rule out a relative increase of 54% in risk of death with shorter duration of DAPT. Furthermore, we have also performed subgroup analysis based on patient characteristics, for example, younger patients (<65 years), older patients (>65 years), diabetic patients, and patients with prior acute coronary syndrome, and showed that early discontinuation of therapy in these subgroups did not increase adverse events. In contrast, Cassese et al. were only able to look at trial characteristics such as trial size, time of randomization, or publication status.

We are also aware of three other recent articles that cover this particular topic but are not eligible for inclusion in our review. Two of these articles were meta-analyses, but their evaluations appear to be less detailed than the current review [29, 30]. The other article provided additional data for one of the trials that was already included, with subgroup analysis suggesting that there are differences between stents [31].

It is notable that in a few of the analyses there was a relatively large contribution by the Valgimigli et al. study [9]. This study had a high event rate compared to other studies as definite or probable stent thrombosis and myocardial infarction was as high as ~4% compared to <1% for all other studies for both outcomes. Possible reasons for the high event rate may be that the patients had higher baseline cardiovascular risk, longer follow-up duration, or because 25% of the patients had received bare metal stents.

The studies by Park et al. [8] and Valgimigli et al. [9] used a landmark analysis design. While this method is employed to estimate in an unbiased way the time-to-event probability for each group assuming the participants are members of that group at the landmark time, this may alter the event rate compared to other trials. The event rate may be affected by the choice of landmark time as events that occurred prior to this would be omitted, which lowers the event rate and reduces power of the study. However, early landmark may lead to misclassification at longer follow-up durations.

There are a few important considerations when interpreting the results of this meta-analysis. The results may not be generalizable to high risk patients such as those with previous stent thrombosis, those with a large number of stents inserted or patients in whom there are technical concerns about the adequacy of stent deployment. Furthermore, while different drug-eluting stents were used in the trials, our results do not evaluate whether there are any differences among them. It is assumed that the randomization process will reduce the likelihood that the type of stent will give rise to imbalance of adverse events. In addition, only clopidogrel and aspirin were considered as dual antiplatelet therapy and our findings do not apply to newer agents such as prasugrel and ticagrelor. We also suggest a cautious interpretation of the results for early discontinuation of dual antiplatelet therapy (<12 months) in ACS because of the likely protective effect of dual antiplatelet therapy on the nonculprit lesions in the coronary circulation.

Our study has several strengths. We only included high quality randomized controlled trials. We included both pooled results and results for different cutoffs for discontinuation of antiplatelet therapy. We also considered both individual outcomes and composite of mortality and MI. In addition, subgroup analysis was performed to examine if differences were observed in older and younger patients, diabetic and nondiabetic patients, and patients with known prior acute coronary syndrome.

Our study had several limitations. Only four studies were included in the meta-analysis and these studies had different definitions of short and long duration of DAPT exposure. Hence, we are unable to make definitive recommendations on the exact duration of DAPT use, although we are reasonably confident that shorter durations do not necessarily lead to increased mortality or myocardial infarction in those with drug-eluting stents.

Secondly, the time of randomization differed in these 4 studies with 2 studies randomized at index PCI (EXCELLENT [6], RESET [7]), 1 study at 1 month after index PCI (PRODIGY [9]), and the last study at 12 months after index PCI (REAL/ZEST-LATE [8]). As a result, patients developing early adverse events (within 30 days and within 6 months, resp.) in the later 2 studies were excluded. Furthermore, in the 2 former studies, patients with recent myocardial infarction (<72 hours and <48 hours, resp.) were also excluded. Therefore, any conclusions derived from pooling these RCTs need to be interpreted with caution before applying them to the general cohort of patients in our day-to-day practice.

Thirdly, different generations of DES and a small proportion of BMS (25% of patients in PRODIGY [9]) were used in the trials included in this analysis. It is possible that there are significant differences between different types of DES that cannot be accounted for in this analysis. Furthermore, current and future innovations in stent design such as bioabsorbable polymer may allow shorter durations of DAPT.

Finally, three of the RCTs were conducted among Koreans and 1 RCT among Caucasians [6–9] and it is unclear if the findings are applicable to other countries or ethnic groups. A recent very small study showed that endothelial function, which plays an important part in vascular tone, regulating blood flow, and platelet function, may be lower in Koreans than Caucasians [32]. Furthermore, genetic polymorphism of CYP2C19 was shown to be an independent predictor of clopidogrel resistance in Korean subjects with coronary artery disease [33]. Although the three Korean studies included low risk patients, in view of their potential genetic influence on endothelial function and clopidogrel resistance, more RCTs involving different populations are pending before we can make generalized conclusions. The findings of our meta-analysis should be cautiously interpreted when considering wider groups of patients in day-to-day clinical practice.

### 4.1. Suggestions for Future Studies

There are at least four other RCTs (ISAR-SAFE trial [34], DAPT trial [35], OPTIMIZE trial [36]), and OPTIDUAL [37]) ongoing which will hopefully shed more light on this challenging topic and guide clinicians to make evidence-based decisions for their patients in the near future. The larger sample sizes will subsequently allow more insight into whether certain subgroups of patients may benefit from longer durations of DAPT.

## 5. Conclusions

The available evidence from meta-analysis of randomized controlled trials shows no significant cardiovascular or mortality benefit with longer durations of DAPT as compared to shorter duration of DAPT. In contrast, a significantly greater risk of major bleeding was seen with longer durations.

## Supplementary Material

The literature search strategy is reported in Appendix 1, while the assessment of study validity is available in Appendix 2. Selection criteria and patient demographics, comorbidities, angiography results, PCI procedural information, study outcomes, and compliance with medications are shown in Appendices 3–7.

## Figures and Tables

**Table 1:** Study design, duration of dual antiplatelet therapy, follow-up, and outcomes evaluated.

Study ID	Design; year of study; country	Definition of short and long duration	Total number in short duration group	Total number in longer duration group	Follow-up duration	Outcomes evaluated
Gwon et al. 2012 (EXCELLENT trial) [6]	Open-label RCT; June 2008–July 2009; South Korea.	Short: 6 months. Long: 12 months.	722	721	1 year after index PCI.	Primary: target vessel failure—composite of cardiac death, myocardial infarction, or target vessel revascularization. Secondary: death from any cause; death or myocardial infarction; stent thrombosis; major bleeding; major adverse cardiocerebral events—composite of death, myocardial infarction, cerebrovascular accident, or any revascularization; safety endpoint—composite of death, myocardial infarction, cerebrovascular accident, stent thrombosis, or thrombolysis In myocardial infarction major bleeding.

Hu and Wang 2012 [10]	Double-blind RCT; September 2008–October 2011; China.	Short: 12 months. Long: >36 months.	88	94	3 years after PCI.	Primary (12 months onwards): target vessel failure, defined as target vessel-related cardiac death or myocardial infarction and target vessel revascularization. Secondary outcomes included stent thrombosis.

Kim et al. 2012 (RESET trial) [7]	Open-label RCT; April 2009–December 2010; South Korea.	Short: 3 months. Long: 12 months.	1,059	1,058	1 year.	Primary: cardiovascular death, myocardial infarction, stent thrombosis, ischemia driven target vessel revascularization, bleeding. Secondary: individual components of primary endpoint plus nontarget vessel revascularization, cerebrovascular accident.

Park et al. 2010 (REAL-LATE and ZEST-LATE) [8]	Open-label RCT; July 2007–September 2008; South Korea.	Short: 12 months. Long: 24 months.	1,344	1,357	Median duration of follow-up 19.2 months after randomization.	Primary: (12 months onwards) myocardial infarction or death from cardiac causes. Secondary: death from any cause; myocardial infarction; cerebrovascular accident; stent thrombosis; repeated revascularization; a composite of myocardial infarction or death from any cause; a composite of myocardial infarction, cerebrovascular accident, or death from any cause; a composite of myocardial infarction, cerebrovascular accident, or death from cardiac causes; and major bleeding.

Valgimigli et al. 2012 (PRODIGY) [9]	Open-label RCT; December 2006–December 2008; Italy.	Short: 6 months. Long: 24 months.	983	987	2 years.	Primary: 30-day to 24-month incidence of death from any cause, nonfatal myocardial infarction, or cerebrovascular accident. Secondary: each component of primary outcome, cardiovascular death, the incidence of stent thrombosis, and bleeding outcomes.

**Table 2:** Rates of thrombotic events in the two groups with different duration of dual antiplatelet therapy.

Study ID	Total number in short duration group	Total number in longer duration group	Stent thrombosis		Myocardial infarction		Stroke		Revascularization		Cerebrovascular death		All-cause mortality		Death and myocardial infarction	
			Short	Long	Short	Long	Short	Long	Short	Long	Short	Long	Short	Long	Short	Long
Gwon et al. 2012 (EXCELLENT trial) [6]	722	721	6 (0.9%) definite 0 (0%) probable	0 definite 1 (0.1%) probable	13 (1.8%)	7 (1%)	3 (0.4%)	5 (0.7%)	Target lesion revascularization 17 (2.4%) Target vessel revascularization 22 (3.1%) Any 43 (6.2%)	Target lesion revascularization 18 (2.8%) Target vessel revascularization 22 (3.2%) Any 43 (6.2%)	2 (0.3%)	3 (0.4%)	4 (0.6%)	7 (1%)	17 (2.4%)	14 (1.9%)

Hu and Wang 2012 [10]	88	94	Incidence 0.2% definite or probable	Incidence 0% definite or probable	Incidence 0.2%	Incidence 1%	NA	NA	NA	NA	NA	NA	Incidence 2.6%	Incidence 2.3%	NA	NA

Kim et al. 2012 (RESET trial) [7]	1,059	1,058	2 (0.2%) definite or probable	3 (0.3%) definite or probable	2 (0.2%)	4 (0.4%)	6 (0.6%)	6 (0.7%)	Target vessel revascularization 31 (3.9%)	Target vessel revascularization 27 (3.7%)	2 (0.2%)	4 (0.4%)	5 (0.5%)	8 (1%)	7 (0.7%)	11 (1.0%)

Park et al. 2010 (REAL-LATE and ZEST-LATE) [8]	1,344	1,357	4 definite (0.3%)	5 definite (0.4%)	7 (0.5%)	10 (0.7%)	4 (0.3%)	9 (0.7%)	Any 26 (2.4%)	Any 36 (3.1%)	NA	NA	13 (10%)	20 (15%)	17 (1.3%)	27 (2.0%)

Valgimigli et al. 2012 (PRODIGY) [9]	983	987	7 definite (0.7%), 15 definite or probable (1.5%), 46 definite or probable or possible (4.7%)	8 definite (0.8%), 13 definite or probable (1.3%), 38 definite or probable or possible (3.9%)	41 (4.2%)	39 (4%)	14 (1.4%)	21 (2.1%)	NA	NA	37 (3.8%)	36 (3.7%)	65 (6.6%)	65 (6.6%)	94 (9.6%)	88 (8.9%)

**Table 3:** Rates of bleeding events in the two groups with different duration of dual antiplatelet therapy.

Study ID	Total number in short duration group	Total number in longer duration group	Thrombolysis in myocardial infarction major bleeding		Gastrointestinal haemorrhage		Intracranial haemorrhage		Total haemorrhage	
			Short	Long	Short	Long	Short	Long	Short	Long
Gwon et al. 2012 (EXCELLENT trial) [6]	722	721	2 (0.3%)	4 (0.6%)	NA	NA	NA	NA	4 (0.6%)	10 (1.4%)
Hu and Wang 2012 [10]	88	94	NA	NA	NA	NA	NA	NA	NA	NA
Kim et al. 2012 (RESET trial) [7]	1,059	1,058	2 (0.2%)	6 (0.6%)	NA	NA	NA	NA	5	10
Park et al. 2010 (REAL-LATE and ZEST-LATE) [8]	1,344	1,357	3 (0.2%)	1 (0.1%)	NA	NA	NA	NA	NA	NA
Valgimigli et al. 2012 (PRODIGY) [9]	983	987	6 (0.6%)	16 (1.6%)	NA	NA	4 (0.4%)	10 (1%)	15 (1.5%)	27 (2.7%)
