# Supplementary material for: Benefits and Harms of Extending the Duration of Dual Antiplatelet Therapy after Percutaneous Coronary Intervention with Drug-Eluting Stents: A Meta-Analysis
Source: ScientificWorldJournal. 2014 Mar 2;2014:794078. doi: 10.1155/2014/794078 (PMC3958680; doi:10.1155/2014/794078)
Supplement: Supplementary file 1 — The literature search strategy is reported in Appendix 1, while the assessment of study validity is available in Appendix 2. Selection criteria and patient demographics, comorbidities, angiography results, PCI procedural information, study outcomes, and compliance with medications are shown in Appendices 3–7. [file 794078.f1.doc]

**Appendix 1:** Study search strategy

Ovid SP EMBASE:
random:.tw.
OR placebo:.mp.
OR double-blind:.tw.

AND

|  | (duration or discontinuation).mp. [mp=title, abstract, subject headings, heading word, drug trade name, original title, device manufacturer, drug manufacturer, device trade name, keyword] |
| --- | --- |

AND

stent.mp. [mp=title, abstract, subject headings, heading word, drug trade name, original title, device manufacturer, drug manufacturer, device trade name, keyword]

AND

|  | antiplatelet.mp. [mp=title, abstract, subject headings, heading word, drug trade name, original title, device manufacturer, drug manufacturer, device trade name, keyword] |
| --- | --- |

PubMed
randomized controlled trial[Publication Type] OR randomized[Title/Abstract] OR placebo[Title/Abstract] AND ("dual antiplatelet therapy"[All Fields] OR "platelet aggregation inhibitors"[MeSH Terms]) AND (("stents"[MeSH Terms] OR "stents"[All Fields] OR "stent"[All Fields]) OR "drug-eluting stents"[MeSH Terms]) AND (duration[All Fields] OR discontinuation[All Fields])

**Appendix 2:** Quality assessments of included randomized controlled trials

| **Study** | **Randomization sequence generation (was the method of generating the random sequence stated?)** | **Allocation concealment (following randomization, was allocation of intervention satisfactorily concealed e.g remote or centralized centre, sealed opaque envelopes)** | **Blinding of participants, personnel and outcome (what type of blinding, and any specific detail on who was blinded)** | **What percentage of patients were lost to follow-up** | **Missing outcome data (were there any prespecified outcomes in the methods section that the authors said they would assess and report, but we were unable to extract the data for)** |
| --- | --- | --- | --- | --- | --- |
| Gwon 2012 | Randomization with a Web-based response system. | Open-label study. | Open-label study.  Blinded outcome adjudication. | 15 lost to follow up (1%). | None. |
| Hu 2012 | NA. | NA. | NA. | NA. | NA. |
| Kim 2012 | Randomization with a Web-based response system. | Open-label study. | Open-label study.  Blinded outcome adjudication. | 31 did not complete follow up (1%). | None. |
| Park 2010 | Computer generated randomization. | Open-label study. | Open-label study.  Blinded outcome adjudication. | 17 did not have complete follow up (0.6%). | None. |
| Valgimigli 2012 (PRODIGY Trial) | Randomization with computer-generated random sequence with blocks size of 4, 8 and 12 and sealed envelopes. | Open-label study. | Open-label study.  Blinded outcome adjudication. | 7 patients lost to follow up (0.4%). | None. |

**Appendix 3:** Patient selection criteria in randomized controlled trials

| **Study** | **Patient selection criteria** |
| --- | --- |
| Gwon 2012 | Inclusion criteria:  At least 1 lesion in a native coronary vessel with a reference diameter of 2.25 to 4.25 mm, stenosis of > 50% by visual estimation, and evidence of myocardial ischemia such as angina, unstable angina, recent myocardial infarction, silent ischemia, a positive functional study, or reversible changes on ECG consistent with ischemia.  Documentation of ischemia not mandatory for lesions with > 75% stenosis.  No limitations on the number of lesions or length of the lesions in effort to reflect real-life clinical practice.  Exclusion criteria:  Myocardial infarction within 72 hours  Severely compromised ventricular dysfunction (ejection fraction< 25%) or cardiogenic shock  Any stent implantation in the target vessel before enrolment  Hemoglobin <10g/dl or platelet < 100 000/microL  creatinine>=265.3micromol/L or dependence on dialysis  Serious hepatic disease  Major bleeding within 3 months or major surgery within 2 months  Allergy to antiplatelet drugs, heparin, stainless steel, contrast agents, everolimus or sirolimus  Elective surgical procedure planned within < 12 months  Life expectancy < 1 year  Significant left main disease defined as stenosis of > 50%  Chronic total occlusion  True bifurcation lesions requiring a planned 2-stent strategy  Active participation in another study |
| Hu 2012 | Inclusion Criteria:  PCI to left main stem  dual antiplatelet therapy with aspirin and clopidogrel at 12 months  Free from events prior to 12 months  Exclusion Criteria:  Adverse events prior to 12 months |
| Kim 2012 | Inclusion criteria:  Patients with stable angina, unstable angina, or acute MI  Diameter stenosis ≥ 50% and reference vessel diameter of 2.5 to 4.0 mm by visual estimation  Elective PCI, eligible for participation  Exclusion criteria:  Prior history of cerebral vascular accidents, peripheral artery diseases, thromboembolic disease or stent thrombosis  Left ventricular ejection fraction < 40%  Lesions with in-stent restenotic lesion, chronic total occlusion, or significant left main disease requiring intervention  Cardiogenic shock  Acute ST-elevation MI within 48 hours after onset of symptoms  Contraindication to antiplatelet agents  Severe hepatic (≥3 times normal values) or renal dysfunction (serum creatinine>2.0 mg/dl) |
| Park 2010 | Inclusion criteria:  Implantation of drug-eluting stents at least 12 months before enrollment  No major adverse cardiovascular event or major bleeding since implantation and were receiving dual antiplatelet therapy at the time of enrollment.  Exclusion criteria:  Contraindication to the use of antiplatelet drug (e.g. a concurrent bleeding diathesis or a history of major bleeding)  Concomitant vascular disease requiring long-term use of clopidogrel  Other established use of clopidogrel (e.g. recent ACS)  Noncardiac coexisting condition resulting in life expectancy of less than 1 year or that might result in noncompliance with the study protocol  Participating in another drug or coronary-device study |
| Valgimigli 2012 (PRODIGY Trial) | Inclusion criteria:  Patients ≥18 years of age with chronic stable coronary artery disease or acute coronary syndromes, including non–ST-elevation and ST-elevation myocardial infarction  At least 1 lesion with a diameter stenosis of ≥50% that was suitable for coronary stent implantation in a vessel with a reference vessel diameter of ≥2.25 mm  No limit for the number of treated lesions, vessels, or lesion length  Exclusion criteria:  Known allergy to acetylsalicylic acid or clopidogrel  Planned surgery within 24 months of percutaneous coronary intervention unless the dual-antiplatelet therapy could be maintained throughout the perisurgical period  History of bleeding diathesis  Major surgery within 15 days  Active bleeding or previous stroke in the past 6 months  Concomitant or foreseeable need for oral anticoagulation therapy  Pregnancy  Life expectancy <24 months  Participation in another trial  Inability to provide informed consent |

**Appendix 4:** Patient demographics, comorbidities and clinical presentation

| Study ID | Patient demographics | | | Comorbidities | | | | | | Clinical presentation | | |
| --- | --- | --- | --- | --- | --- | --- | --- | --- | --- | --- | --- | --- |
|  | Mean age | % Male | % Current smoker | Prior MI | Prior congestive cardiac failure or left ventricular dysfunction | Left ventricular ejection fraction (%) | Prior diabetes | Prior cerebrovascular accident | Prior renal impairment | Chronic stable angina | ACS | STEMI |
| Gwon 2012 (EXCELLENT trial) | 63 | 65% | 27% | 5% * | 0.7% | 61 | 38% | 7% | 1% | 48% | 48% | 3% |
| Hu 2012 | NA | NA | NA | NA | NA | NA | NA | NA | NA | NA | NA | NA |
| Kim 2012 (RESET trial) | 62 | 64% | 24% | 2% | 12% | 64 | 29% | NA | NA | 45% | 55% | NA |
| Park 2010 (REAL-LATE and ZEST-LATE) | 62 | 70% | 31% | 4% | Ejection fraction: 59% | 59 | 26% | 4% | NA | 38% | 62% | 11% |
| Valgimigli 2012 (PRODIGY) | 68 | 77% | 24% | 27% | Ejection fraction: 53% | 53 | 24% | 4% (cerebrovascular events or transient ischemic attack. | NA | 26% | 74% | 33% |

* groups differ significantly

**Appendix 5:** Procedural information on percutaneous coronary intervention

| Study ID | Procedural data | | | | | | | | Procedural success |
| --- | --- | --- | --- | --- | --- | --- | --- | --- | --- |
|  | Type of stent | Number of stents per patient | Stents per lesion | Mean stent length (mm) | Mean stent caliber (mm) | % American College of Cardiology/American Heart Association classification type B2/C lesions | % of intravascular ultrasound | % use of GIIb/IIIa inhibitors |  |
| Gwon 2012 (EXCELLENT trial) | 75% EES 25% SES | 1.6 | 1.2 | 28 | NA | 51% (n=991) | 43% (n=627) | 1.7% (n=24) | 99% |
| Hu 2012 | Drug eluting stent. | NA | NA | NA | NA | NA | NA | NA | NA |
| Kim 2012 (RESET trial) | 50% E-ZES vs 21% R-ZES, 14% SES or 15% EES | NA | NA | 23 | 3.2 | 69% (n=1,842) | NA | 2% (n=41) | 99.9% |
| Park 2010 (REAL-LATE and ZEST-LATE) | 57% ZES/  24% PES/  18.5% SES  0.5% Others | 1.8 * | 1.3 | 31 | NA | 79% (n=2955) | NA | NA | 100% |
| Valgimigli 2012 (PRODIGY) | 25% EES/ 25% PES/ 25% ZES/ 25% BMS | 1.9 | NA | 30 median | 3.0 median | 66% (n=1306) | NA | NA | NA |

* calculated

**Appendix 6:** Angiography results

| Study ID | Angiogram data | | | | | | | |
| --- | --- | --- | --- | --- | --- | --- | --- | --- |
|  | % multivessel disease | % bifurcation lesions | % CTO | % LMS treated | % LAD treated | % LCx treated | % RCA treated | % graft treated |
| Gwon 2012 (EXCELLENT trial) | 52% (n=750) | 11% (n=209) | 3% | Excluded | 66% | NA | NA | NA |
| Hu 2012 | NA | NA | NA | 100% | NA | NA | NA | NA |
| Kim 2012 (RESET trial) | 43% | NA | NA | NA | 53% | 20% | 27% | NA |
| Park 2010 (REAL-LATE and ZEST-LATE) | 48% | 12% | 11% | 2.6% | 49% | 19% | 29% | NA |
| Valgimigli 2012 (PRODIGY) | 65% | NA | NA | 6% | 53% | 32% | 36% | 1.3% |

**Appendix 7:** Study outcomes, follow-up, compliance with medications and dosage of antiplatelet therapy

| Study ID | Definition of outcomes | | Follow up-information | | Compliance with medication | | | Dose of antiplatelet agents used |
| --- | --- | --- | --- | --- | --- | --- | --- | --- |
|  | Definition of thrombotic outcomes | Definition of bleeding outcomes | Follow-up duration | Methods used | % DAPT at discharge | % DAPT at end of short duration | % DAPT at end of long duration |  |
| Gwon 2012 (EXCELLENT trial) | ARC/ Imaging + neurologist. | Thrombolysis In Myocardial Infarction criteria. | 12 months. | Clinical follow-up. | 97.7 | 71.2% | 93.2% | Aspirin: 100 - 200mg  Clopidogrel: 75mg |
| Hu 2012 | NA | NA | 3-years. | NA | NA | NA | NA | Aspirin: 100 mg  Clopidogrel: 75mg |
| Kim 2012 (RESET trial) | ARC. | Thrombolysis In Myocardial Infarction criteria. | 12 months. | Clinic visit or telephone interview. | NA | 94.1% | 100% | Aspirin: 100mg  Clopidogrel: 75mg |
| Park 2010 (REAL-LATE and ZEST-LATE) | ARC/ Imaging + neurologist. | Thrombolysis In Myocardial Infarction criteria. | Median duration 19.2 months. | Clinical follow up. | 99.7% | NA | 90% at 12 months  80% at 24 months | Aspirin: 100 – 200mg  Clopidogrel: 75mg |
| Valgimigli 2012 (PRODIGY) | ARC/ Imaging + neurologist. | BARC/ Thrombolysis In Myocardial Infarction criteria. | 2 years. | Follow-up study visits. | At 30 days, 100% | 91% at 6 months | 97% at 24 months | Aspirin: 100 - 200mg  Clopidogrel: 75mg |
